# Development and promotion in translational medicine: perspectives from 2012 sino-american symposium on clinical and translational medicine

**DOI:** 10.1186/2001-1326-1-25

**Published:** 2012-10-24

**Authors:** Mengjia Qian, Duojiao Wu, Ena Wang, Francesco M Marincola, Wei Wang, William Rhodes, Michael Liebman, Chunxue Bai, Ching-Wan Lam, Gyorgy Marko-Varga, Thomas E Fehniger, Roland Andersson, Xiangdong Wang

**Affiliations:** 1 Biomedical Research Center, Shanghai, China; 2 Department of Pulmonary Medicine, Fudan University School of Medicine, Zhongshan Hospital, Shanghai, China; 3 Infectious Disease and Immunogenetics Section (IDIS), Department of Transfusion Medicine, Clinical Center and Center for Human Immunology (CHI), NIH, Bethesda, MD, USA; 4 School of Medical Sciences, Edith Cowan University, Perth, Australia; 5 Beijing Municipal Key Laboratory of Clinical Epidemiology, Graduate University of the Chinese Academy of Sciences, Beijing, China; 6 Becton Dickinson and Co, Franklin Lakes, New Jersey, USA; 7 IPQ Analytics, LLC and of Strategic Medicine, Inc, Strategic Medicine, BV, The Hague, NL; 8 Department of Pathology, The University of Hong Kong, Hong Kong, China; 9 Department of Surgery, Clinical Sciences, Medical Faculty, Lund University, Lund, Sweden

## Abstract

**Background:**

Clinical translational medicine (CTM) is an emerging area comprising multidisciplinary research from basic science to medical applications and entails a close collaboration among hospital, academia and industry.

**Findings:**

This Session focused discussing on new models for project development and promotion in translational medicine. The conference stimulated the scientific and commercial communication of project development between academies and companies, shared the advanced knowledge and expertise of clinical applications, and created the environment for collaborations.

**Conclusions:**

Although strategic collaborations between corporate and academic institutions have resulted in a state of resurgence in the market, new cooperation models still need time to tell whether they will improve the translational medicine process.

## Introduction

Clinical translational medicine (CTM) is an emerging area comprising multidisciplinary research from basic science to medical applications and entails a close collaboration among hospital, academia and industry [1]. CTM is to bridge the divide between health informatics ‘bench research’ and the application of informatics in clinical and health care settings [2]. The critical care community is beginning to adopt an increasingly translational approach to research, drug development and early-phase clinical trials [3].

The 2012 Sino-American Symposium on Clinical and Translational Medicine (SAS-CTM) served as one of the milestone conferences on CTM and was organized by Chinese Academy of Engineering, Chinese Academy of Medical Sciences, The U.S. National Institutes of Health Clinical Center, and Global MD Organization. As a part of 2012 SAS-CTM, session seven, Project Development and Promotion in Translational Medicine, was co-chaired by Dr. Roland Anderson, Professor of Department of Surgery, Clinical Sciences,Medical Faculty, Lund University and Dr. Xiangdong Wang, Professor of Medicine and Director, Biomedical Research Center, Fudan University Zhongshan Hospital. The Session focused on new models for project development and promotion in translational medicine.

## Findings

### Current situation of translational medicine

Translational medicine is a growing and emerging area that integrates basic, social, clinical and political science together to efficiently improve patient care and outcomes. However, we should know there are lessons to be learned about the best approaches for the promotion of translational medicine [4]. So, we need to have a general knowledge of the current situation of translational medicine first.

Dr. Ena Wang, Senior Staff Scientist, Director of Molecular Science of DTM-CC and Associate Director of Center for Human Immunology of NIH, presented a system biology approach in current translational medicine application. She shared her studies that they used translational medicine methodology to characterize the human immune responsiveness in both healthy individuals and the cancer patients which were adopting immunotherapies. She summarized the process from observing clinical phenomenon to generating hypothesis and finishing data interpretationwhich let us have a direct view of what is translational research.

Mr. William Rhodes, Senior Vice President of Corporate Strategy and Development, Becton Dickinson & Company, described the current role of corporate-academic research partnerships in translational medicine, and what might be done to improve it. The traditional model of drug development is to focus on ‘investigator-centric’ research; that is, healthcare companies fund investigators to undertake clinical research of the manufacturers’ products. In most nations, there are regulations that govern the ability of corporations to fund basic research in hospitals and academic settings. This potentially creates a schism between the rapid translation of new medical discoveries to medical practice and the source of funding that might enable it. Meanwhile, health systems around the world are experiencing increasing pressure to treat more patients in shorter times, with an eye towards cost control as well as efficacy, and patients are advocating for more rapid availability of new treatment modalities.

This approach does not promote the early study and testing of novel therapeutic concepts based on contemporaneous and new experimental data – it can, in fact, impede the movement of new treatments and diagnostics from laboratory to patient. Given the rapid and important advances in basic cellular research, coupled with single cell genomics, proteomics and the like, it seems likely that important new discoveries, which can be applied to the effective diagnosis and treatment of disease, may languish as they proceed along a protracted timeline, or worse yet, never make it from lab to patient. Industry must, then, adopt a more progressive model, working with academia and regulators, to foster more rapid translation of laboratory advances to clinical practice, in order to become more relevant and competitive. Without this, the schism mentioned above will continue to widen, stalling the development of new effective diagnostic tools and disease treatments.

The reports from these two professors gave us a general idea of the current situation of translational medicine, but we still need to select the right collaboration model and the developing way of our translational program.

### New collaboration models

#### Collaboration between academia and industry

How might we interact among stakeholders to optimally integrate health care delivery, academic research and the healthcare industry to improve the efficacy of truly translational medicine? What are the challenges?? In its simplest form, the solution lies in closer communication and integration among the various players, including product developers, academic researchers, healthcare providers and governments. There is a new model emerging to address this. Targeted drug development is one example of success in clinical and translational medicine. Several countries are now promoting the integration of their previously separated health and academic sectors. For example, in the U.K., Germany, Australia, Brazil, and Singapore, institutes have been established that create a strong link with industry and cut across various areas of science and medical practice. Meanwhile, academic institutions and government research centers are emerging as true partners with industry in the drug discovery and evaluation process. An example is the proposed National Center for Advancing Translational Science (NCATS) at the US NIH, which will become a key catalyst, integrator and leader to insure the interface of government, academic institutions and industry to promote translational medicine. In addition, pharmaceutical, biotech and other healthcare product developers are increasingly entering milestone-based funding agreements with academia and clinical investigators, sharing the risk of product development at a much earlier stage, in order to more rapidly move scientific discoveries to the treatment of patients and ultimately, commercialization.

#### Partnership business models

**Figure 1:**
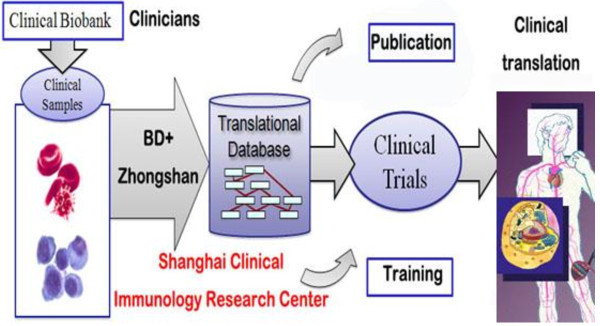
**Success Story**: **Shanghai clinical immunology research center.** It has supported molecular immunology training program developed collaboratively between BD and Shanghai Zhongshan Hospital partners. It has proven very popular with both clinical fellows and industry, and acted as a catalyst for greater industry engagement at translational medicine.

While there are various forms and structures of partnership business models, one that has proven successful has the product development organization (company) establishing a more integrated and flexible external network of academic experts. Such an example is Chorus, an autonomous division of Eli Lilly and Company [5], that is an early phase drug development group that cost-effectively advances candidate molecules from discovery through clinical proof of concept (PoC). In the seven years since its inception, Chorus has advanced two dozen molecules into development from discovery through early phase clinical study in patients. Another model calls for industry sharing its resources with multiple medical researchers and clinicians in Centers of Excellence (COEs), an example of which is the Shanghai Clinical Immunology Research Center [6], founded by Becton Dickinson & Co. (BD) and Zhongshan Hospital Fudan University. This COE allows researchers to share the most advanced and specialized laboratory equipment and clinical samples (Figure 1). Another approach is to develop broad and flexible institution-wide collaborations, a representative of which is Pfizer’s Center for Therapeutic Innovation (CTI) [7], an entrepreneurial research unit at Pfizer dedicated to establishing global partnerships between Academic Medical Centers (AMCs) and Pfizer to focus on translational medicine. CTI laboratory staff includes Pfizer employees working side-by-side with leading basic and translational science investigators and post-docs from the AMCs. CTI established partnerships with 20 leading academic medical centers across the United States and supports collaborative projects from four dedicated labs in Boston, New York City, San Francisco, and San Diego. CTI received over 300 proposals from its first round of applications, and to-date has selected approximately 20 programs to initiate in partnership with the proposing PIs. Funded programs span a broad range of therapeutic areas, including oncology, inflammation, infectious disease, cardiovascular and metabolic disease.

### Trends of developing translational medicine

In this conference, our major focus is to develop the multidisciplinary approach and academic/industry partnership in this translational medicine area in order to improve our understanding of diseases, and enhance cost-effective decision making in exploratory development [8]. During the conference, two professors shared their successful experiences in translational medicine. Dr. Michael N. Liebman, CEO of Strategic Medicine Inc, described the clinical and translational medicine treatment in rare diseases and pediatrics that are being carried out in the United States and extended to Europe and potentially China [9]. Dr. Wei Wang, Professor of School of Medical Sciences, Edith Cowan University, Perth, Australia, and he is also the Dean of School of Public Health and Family Medicine, Capital Medical University and Professor of the Chinese Academy of Sciences, Beijing, China, described the translational medicine research in traditional Chinese medicine, mainly focusing on suboptimal-health status ‘a new health dimension for translational medicine” [10,11]. These two successful cases let us have full confidence in the development of translational medicine and get an idea of the developing trend of translational medicine.

#### From biobanking to precision medication

Dr. Gyorgy Marko-Varga, Professor of Lund University, described the biobanking facility in his hospital. To set up a specimen bank requires the collection of specimens while strictly following good clinical practice principles. The specimens collected have to be well identified and kept in strict environmental controlled facilities from the time of collection to the final storage [12]. Though the establishment of biobankis not easy, the benefit to clinical research is obvious such as promoting precision medication. Professor described his studies in individualized medication from screening drug delivery to gradual realization of precision medicine, thus ensuring patients the best and the most effective drugs in the early stage of diseases. We can obtain many specimens from biobank with related information that can be used in clinical translational research, thus realizing precision medicine which provides good prospects for establishing rational use of drugs, improving curative effects and reducing adverse reactions.

#### From bioinformatics to dynamic biomarker network

Some complex diseases are not caused by the maturation of single gene or single protein. It is an integration of networks, so we can’t diagnose these diseases depending only based on one or several genes and proteins. Numerous genetic and genomic datasets or proteomic datasets related to complex diseases have been made available during the last decade [13].

Dr. Ching-Wan Lam, Professor of the University of Hong Kong, described his studies of omics-based medicine. He explained the concept of metabolomics. Metabolomics can be defined as the field of science that deals with the measurement of metabolites in an organism and studies the physiological processes and reactions to various pathological stimuli. Metabolomic analysis can be translated into clinical medicine in terms of disease identification, early disease detection and screening through metabolomic analysis of human biofluids.

A number of methodologies and computational programs have been developed to integrate selected proteins into the knowledge-based networks via the combination of genomics, proteomics and bioinformatics [14]. Dr. Francesco Marincola, Chief of the Infectious Disease and Immunogenetics Section in the Department of Transfusion Medicine at the Clinical Center of the National Institutes of Health, reported his studies in finding network biomarkers in the metastatic cancer model. Bioinformatics analysis can provide a valuable molecular basis for systematic interpretation of the mechanism underlying metastasis where potential protein markers could be characterized [15].

The studies of these two professors made us have full confidence in the promotion of translational medicine in this field.

#### From internet of things to telemedicine

Internet of Things is one of the major communication advances in present time that links the internet with everyday sensors and working devices for an all –IP based architecture [16]. Internet of Things in Medicine is broadly recognized as a potentially important tool for transforming medical care and public health [17]. Internet of Things in Medicine includes many kinds of sensors within medical devices, combines the Internet of Things with the current internet, thus perfectly achieving the integration of hospitals, patients and medical devices and facilitating a totally new modern medical mode.

Telemedicine is the application of telemetry in the practice of medicine. To a comprehensive definition is in usage of exchanged medical information through electronic networks to improve a patient’s education, healthcare provider’s education, and patient care [18]. Dr. Chunxue Bai, Director of Shanghai Respiratory Research Institute, described his telemedicine mode to us--Cell Phone Based Remote Monitoring System for Lung Function. This invention can improve COPD management, asthma management and ICU ventilator management. It will let people diagnose diseases earlier, receive treatments earlier thus reducing the risks and even saving lives.

Our current complex health-care systems are fragmented, and their functioning, both in terms of efficiency and quality, are plagued by multiple discontinuities. But E-Health has the potential to ease transitions between the many settings and stakeholders of healthcare [19]. So, application of Internet of Things in Medicine is shall to be more benefit to people, it will be a developing trend in translational medicine in future.

## Conclusions

**Figure 2:**
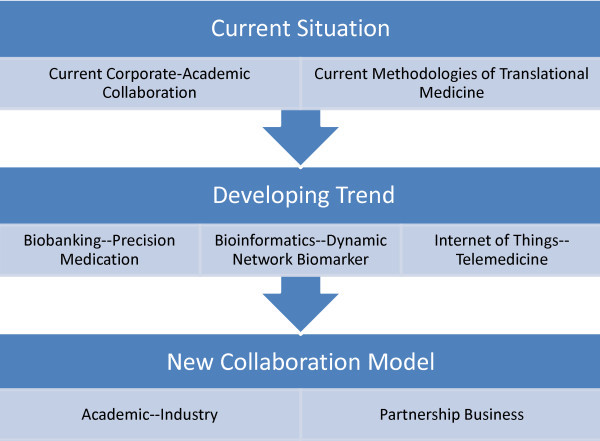
**Translational medicine is a growing and merging area to integrate basic, social, clinic and political sciences together to efficiently improve patient care and outcomes.** We should have a general idea of the current situation, developing trend and new collaboration models of translational medicine thus we can find a right way to promote translational program.

On the basis of these professors’ reports, we have had a general idea of the current situation, the new collaboration models and the future developing trend of the translational medicine, shown in Figure 2. The conference stimulated the scientific and commercial communication of project development between academies and companies, shared the advanced knowledge and expertise of clinical applications, and created the environment for collaborations. It was also a golden opportunity for both academic and company scientists to explore the potential for further collaborations and support for each other. Although strategic collaborations between corporate and academic institutions have resulted in a state of resurgence in the market, new cooperation models still need time to tell whether they will improve the translational medicine process.

## Competing interests

The authors declare that they have no competing interests.

## Authors’ contribution

MJQ and DJW drafted the manuscript, EW, FMM, WW, WR, ML, CXB, CWL, GMV, TEF, RA,XDW edited and reviewed. All authors read and approved the final manuscript.
